# ARE THERE DIFFERENCES IN CHRONIC PAIN AFTER LAPAROSCOPIC INGUINAL HERNIA REPAIR USING THE TRANSABDOMINAL TECHNIQUE COMPARING WITH FIXATION OF THE MESH WITH STAPLES, WITH GLUE OR WITHOUT FIXATION? A CLINICAL RANDOMIZED, DOUBLE-BLIND TRIAL

**DOI:** 10.1590/0102-672020220002e1670

**Published:** 2022-09-09

**Authors:** Maurício Andrade Azevedo, Guilherme Blattner Torres de Oliveira, Carlos Alberto Malheiros, Sergio Roll

**Affiliations:** 1 Universidade Federal de São Paulo, Gastrosurgery – São Paulo (SP), Brazil; 2 Faculty of Medical Sciences of Santa Casa of São Paulo, General Surgery – São Paulo (SP), Brazil.

**Keywords:** Hernia, Inguinal, Chronic Pain, Laparoscopy, Recurrence, Hérnia Inguinal, Dor Crônica, Laparoscopia, Recidiva

## Abstract

**BACKGROUND:**

Regarding postoperative pain, it remains unclear whether non-fixation of the polypropylene prosthesis in transabdominal preperitoneal inguinal hernia repair produces the same outcomes as mesh fixation with glue or tackers. In addition, hernia recurrence is another aspect to be assessed in the comparison between non-fixation and mesh-fixation techniques (tackers and glue).

**AIMS:**

This study aimed to evaluate the incidence, quality of pain, and recurrence in patients undergoing laparoscopic inguinal hernioplasty (transabdominal preperitoneal) technique, comparing the fixation of the mesh with tackers versus with glue versus without fixation.

**METHODS:**

This is a prospective, double-blind study in which 63 patients presenting with primary unilateral inguinal hernia underwent laparoscopic transabdominal preperitoneal inguinal hernia repair and were randomized into three groups: no mesh fixation (n=21), mesh tacked (n=21), and mesh fixed with fibrin glue (n=21). Patients also responded to questionnaires in order to assess pain and pain quality and were followed up for 2 years.

**RESULTS:**

Neither mesh-fixation nor non-fixation techniques were found to affect postoperative chronic pain (p=0.535), but patients undergoing tacker fixation reported more pain descriptors (p=0.0021) and a higher pain index (p=0.002) on the McGill scale in the first 15 postoperative days (T0 and T1). No hernia recurrences were observed.

**CONCLUSIONS:**

Both mesh-fixation techniques (tackers and glue) used with the transabdominal preperitoneal approach did not influence the onset of inguinodynia, but tacker fixation was more likely to increase patient sensitivity to pain. Mesh placement without fixation produced the same pain and recurrence outcomes as mesh-fixation techniques. Also, no recurrence was observed in patients without mesh fixation in this study. Consequently, it has become an alternative therapy deserving consideration for hernia repair.

## RESUMO

**RACIONAL:**

Em relação à inguinodinia, há que se perguntar se a não fixação da tela pela técnica da hernioplastia inguinal videolaparoscópica transbadominal pré-peritoneal teria os mesmos resultados em relação à fixação de telas com cola ou grampos. Além disso, a recorrência de hérnia é outro aspecto a ser avaliado na comparação entre as técnicas de não fixação e de fixação com tela (grampos e cola).

**OBJETIVOS:**

Avaliar a incidência, qualidade da dor e recorrência em pacientes submetidos à técnica de hernioplastia inguinal laparoscópica (transbadominal pré-peritoneal), comparando a fixação da tela com grampos vs. com cola vs. sem fixação.

**MÉTODOS:**

Este é um trabalho prospectivo, duplo-cego, em que 63 doentes portadores de hérnia inguinal submetidos à hernioplastia inguinal videolaparoscópica pela técnica transbadominal pré-peritoneal foram randomizados em três grupos: no primeiro a tela não foi fixada; no segundo foi fixada por grampos; e no terceiro foi fixada com cola. Estes pacientes foram submetidos a questionários para avaliação de dor, sendo acompanhados por dois anos.

**RESULTADOS:**

O método de fixação da tela, assim como a não fixação dela não interferiu no aparecimento da dor crônica pela Escala Visual Analógica; porém, os que foram submetidos à fixação por grampos tiveram mais descritores e índice de dor pela escala de McGill. Não foram observadas recidivas herniárias.

**CONCLUSÕES:**

O método de fixação da tela na técnica transbadominal pré-peritoneal não influencia no aparecimento da inguinodinia. A não fixação teve os mesmos resultados em termos de dor e recidiva, tornando-se alternativa terapêutica a ser considerada, pois não acarretou recidivas.

## INTRODUCTION

Inguinal hernioplasty is among the most common types of procedures performed by general surgeons^28^. It is estimated that approximately 20 million hernioplasties are performed worldwide^19,20^ and in Brazil in the year of 2019, 122.631 hernioplasties were performed^8^ in the public health system.

The use of meshes in the surgical treatment of inguinal hernias has led to decreased rates of recurrence. Consequently, chronic pain, also known as inguinodynia, has become the main postoperative complication^14,26^.

Inguinodynia, which can be defined as moderate- to high-intensity pain lasting for over 3 months postoperatively, has an estimated risk of 10–12%. In addition, the incidence of severe pain is reported to range from 0.5 to 6%, with complex and multifactorial causes^14,26^. The major etiologies of chronic pain comprise hernia recurrence, neuropathic causes (direct nerve injuries or perineural injuries), and non-neuropathic causes (scar tissue produced by mesh fixation or foreign-body reaction to the mesh^5,11,17,23,26,33^.

In this respect, videolaparoscopic hernioplasty offers several advantages, including lower postoperative pain and faster return to usual activities^3,9^. According to the guidelines of the European Hernia Society for both the transabdominal preperitoneal (TAPP) and laparoscopy technique that is totally extraperitoneal (TEP), a mesh can be stapled or glued, or not fixated as long as the hernia defect does not exceed 3 cm in size^3,4,7,9,10^.

Many studies have not shown differences in pain and recurrence outcomes between these two techniques^2,20,27^. However, there is a higher number of studies demonstrating that mesh fixation is not necessary in the TEP technique^16,19,20,25,29,34^, while there are only a few studies on TAPP hernia repair without mesh fixation^6,30^.

Regarding postoperative pain, the question is whether non-fixation of the polypropylene mesh in videolaparoscopic TAPP inguinal hernioplasty produces the same outcomes as mesh fixation with fibrin glue or tackers^26^. In addition, further studies on both the quantity and quality of patient-reported pain are needed to establish the differences between tacked and glued meshes, if at all. In addition, hernia recurrence rates following TAPP procedures without mesh fixation should be compared to mesh fixation with tackers or glue.

The aim of this study was to evaluate the incidence, quality of pain, and recurrence in patients undergoing laparoscopic inguinal hernioplasty (TAPP) technique, comparing the fixation of the mesh with tackers versus with glue versus without fixation.

## METHODS

This study was approved by the Ethics and Research Committee of the Transplant Hospital Dr. Euryclides de Jesus Zerbini CAAE (Presentation Certificate for Ethical Appreciation: 61243016.1.0000.0091 and approval of Plataforma Brasil number: 1.829.953 and the Committee of Randomized Trials — ReBEC [Brazilian Registry of Clinical Trials]: Trial Registration number 6d7twh, date of registration: September 16, 2019 and URL: http://www.ensaiosclinicos.gov.br/rg/RBR-6d7twh/).

This is a prospective, double-blind, randomized trial conducted between November 2016 and November 2019 where 125 patients were attended in the ambulatory of the general surgery from the hospital presenting with inguinal hernia complains. Inclusion criteria were as follows:

- adults aged between 18 and 75 years;
- having Nyhus type 2 or 3 unilateral inguinal hernias; and
- rated as Class I or II in the physical status classification System of the American Society of Anesthesiology (ASA).

Only 63 patients were selected for this study. The remaining 62 were excluded because they did meet the inclusion criteria or have one of the following conditions:

- adults aged under 18 and older than 75 years;
- having bilateral inguinal, incarcerated, strangulated hernia, or undergone previous surgery in the inguinocrural region, the right iliac fossa and hypogastrium, or prostate surgery;
- chronic analgesic, corticosteroid, antidepressant, and anxiolytic users;
- those rated as ASA Classes III, IV, and V; or
- no consent to randomization.

A total of 63 sequential patients presenting with primary unilateral inguinal hernia underwent TAPP inguinal hernia repair and were operated in this same hospital. The procedure was performed by the same surgical team, and this trial method was performed in accordance with the relevant guidelines and regulations and an informed consent was obtained from all patients after explaining the purpose of the study as well as free consent to refuse.

Patients were randomized into three groups of 21 individuals. At the time of surgery, a randomization application (Random®) was used to allocate them into three different groups as mesh-fixation groups: fibrin glue (21), tackers (21), or non-fixation (21).

### Surgical technique

After induction of anesthesia, patients were placed in the Trendelenburg position and in contralateral rotation to the hernia site. Subsequently, with a Veress needle pneumoperitoneum was created, an 11-mm trocar was placed in the umbilical region, and two 5-mm trocars were inserted into the lateral borders of the rectus abdominis muscle. The parietal peritoneum was then opened, and the main anatomical landmarks were identified (lower epigastric vessels, iliac vessels, spermatic cord, pectineal ligament, rectus abdominis muscle) followed by dissection and reduction of the hernial sac and lipoma removal when necessary. In nerve topography, extraperitoneal fat was preserved in order to protect the nerves. Next, an anatomical review of both the entire myopectineal orifice and all anatomical landmarks and references was made. Subsequently, a heavyweight polypropylene mesh (100 g/m^2^) of at least 15 cm by 12 cm was introduced and placed in the dissected preperitoneal space.

After a mesh-fixation method, or no mesh fixation, was chosen, according to the group, the peritoneum was then sutured with 2–0 polyglactin, and CO_2_ aspiration was performed in the preperitoneal space. Then the pneumoperitoneum was desuflated, the trocars were removed, followed by muscle and aponeurotic closure with 0 polyglactin, and skin suturing with simple stitches. In fibrin glue group (FGG), the mesh was fixated with instillation of n-butyl-2-cyanoacrylate (Glubran 2, GEM®, Italy) tissue adhesive on the pectineal ligament, rectus abdominis muscle, and laterally of the inferior epigastric vessels 3 cm above iliopubic tract. In tacker group (TG), tacker fixation of the mesh was performed using an Absorbatack® stapler (Covidien-Medtronic®, Minneapolis, USA) onto the pectineal ligament, rectus abdominis muscle, and laterally of the inferior epigastric vessels 3 cm above iliopubic tract. The control was non-fixation group (NFG).

Mesh was placed covering the pectineal ligament, the rectus abdominis muscle, and laterally of the inferior epigastric vessels without fixation.

With the purpose for quantifying and qualifying postoperative pain in the first postoperative day (T0), patients were evaluated through physical examination by a surgeon who had not participated in the surgery and did not know whether the mesh had been fixated, or which fixation method had been used (no access to operate notes). To this end, two questionnaires were responded to: one containing a multidimensional classification of pain with verbal, numeric, and face scales (Visual Analog Scale — VAS), and the other being the McGill pain index and pain quality questionnaire^26^.

Postoperative follow-up took place between Day 7 and Day 15 (T1), after 3 and before 6 months (T2), after 1 year (T3), after 1 year and 6 months (T4), and after 2 years (T5) following the surgery. For this, a third surgeon who had not participated in the surgery or evaluated the patient in the first postoperative period was chosen to evaluate patients through physical examination looking for recurrence and any complication and with a questionnaire containing a multidimensional classification of pain using verbal, numeric, and face scales (VAS) and the McGill pain questionnaire.

Regarding the period of pain, inguinodynia was defined as persistent pain lasting over 3 months. Pain was stratified according to a multidimensional pain classification scale, comprising verbal, numeric, and face scales (VAS: absence of pain=0 or presence of pain=1–10)^24^ and the McGill pain questionnaire^26^, including calculation of the number of pain descriptors (range 1–20) and of a pain index (range 1–78).

### Statistical analysis

To estimate the sample, the pain score was used as the main criterion. At minimum, a sample of 21 patients per group (total of 63) is required to detect differences in pain averages, assessed over seven time periods (before and 15–30, 90–180 days, 1 year, 1 year and 6 months, and 2 years after surgery), with an 88.9% power in the analysis of variances, with repeated measures (F-test with Geisser-Greenhouse correction), at a significance level of 5%. For this purpose, a sample size effect of 0.54 between groups, 1.86 for time, and a 0.50 autoregressive correlation structure was accepted. Both Friedman and Wilcoxon signed-rank tests were used to find out whether the treatments were effective. Also, Kruskal-Wallis and Mann-Whitney U tests, in addition to the chi-square test, were performed to calculate the difference, if at all, between the groups. A level of 0.05 (p<0.05) was defined for this study. In this statistical analysis, the following software programs were used: SPSS version 20, Minitab 16, and Excel Office 2010.

## RESULTS

**Figure 1:**
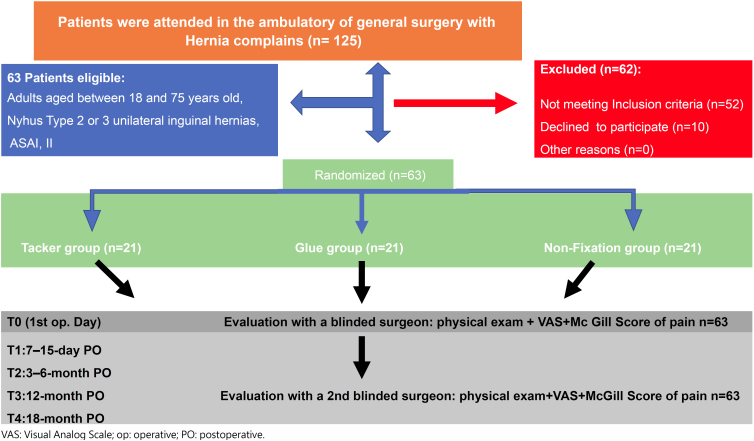
Flow randomization and follow-up.

At the beginning of this study, 125 patients were sequentially evaluated in the ambulatory of the general surgery, but 52 patients did not fit one or more of the inclusion criteria and 10 did not accept to be part of the randomization (Figure 1). The 63 patients in this study were rated following the European Hernia Society (EHS) criteria^33^. One of them had an L1 hernia, 46 had L2, and 6 had L3 hernias. In addition, only two patients were classified as having M1 hernias, while eight had M2 hernias. No patient was found to have M3 hernias (Table 1).

**Table 1:** Patient distribution following the European Hernia Society classification^33^ (n=63).

European Hernia Society classification		
Size (cm)	Location	
	Medial	Lateral
Up to 1.5	2	1
1.5–3.0	8	46
>3.0	0	6

The characteristics of our groups are summarized in Table 2. Regarding characteristics, 61 (96.83%) were male while 2 (3.17%) were female; 84% of hernia defects were indirect (n=53) whereas 16% had direct hernias (n=10); 85.7% (n=54) had hernia defects of up to 3 cm in size while 4.8% had defects no larger than 1.5 cm (n=3) and 9.5% (n=6) had defects larger than 3 cm in size. The rate of seroma formation was 7.9% (n=5). These patients underwent conservative treatment and healed in up to 6 months, while 1 (4.8%) had an intraoperative complication.

**Table 2:** Group comparison for qualitative covariable distribution.

		Non-fixation		Fibrin Glue		Tackers		Total		p-value
		n	%	n	%	n	%	n	%	
Intraoperative complication	No	21	100	21	95.2	21	100	63	98.4	0.362
	Yes	0	0.0	1	4.8	0	0.0	1	1.6	
Seroma	No	21	100	21	95.2	21	100	63	92.1	0.805
	Yes	2	9.5	1	4.8	2	9.5	5	7.9	
Type of hernia	Direct (M)	2	9.5	3	14.3	5	23.8	10	15.9	0.435
	Indirect (L)	19	90.5	18	85.7	16	76.2	53	84.1	
Gender	Female	2	9.5	0	0.0	0	0.0	2	3.2	0.127
	Male	19	90.5	21	100	21	100	61	96.8	
Defect size (cm)	Up to 1.5	1	4.8	0	0.0	2	9.5	3	4.8	0.558
	Up to 3	18	85.7	18	85.7	18	85.7	54	85.7	
	>3	2	9.5	3	14.3	1	4.8	6	9.5	

M: Medial; L: Lateral.

No chronic pain was found in any of the groups. Likewise, no statistical difference in pain on the VAS pain scale was observed between the three groups at any point during postoperative follow-up (Table 3).

**Table 3:** Group comparison on the visual analog scale pain scale per follow-up period.

VAS		Average	Median	Standard deviation	n	CI	p-value
T0	Non-fixation	1.90	1	1.51	21	0.65	0.535
	Glue	1.57	1	1.36	21	0.58	
	Tackers	2.38	2	1.77	21	0.76	
T1	Non-fixation	0.48	0	0.68	21	0.29	0.565
	Glue	0.38	0	0.67	21	0.29	
	Tackers	0.67	0	1.24	21	0.53	
T2	Non-fixation	0.05	0	0.22	21	0.09	0.317
	Glue	0.00	0	0.00	21	–	
	Tackers	0.00	0	0.00	21	–	
T3	Non-fixation	0.00	0	0.00	21	–	1,000
	Glue	0.00	0	0.00	21	–	
	Tackers	0.00	0	0.00	21	–	
T4	Non-fixation	0.00	0	0.00	21	–	1,000
	Glue	0.00	0	0.00	21	–	
	Tackers	0.00	0	0.00	21	–	
T5	Non-fixation	0.00	0	0.00	21	–	1,000
	Glue	0.00	0	0.00	21	–	
	Tackers	0.00	0	0.00	21	–	

VAS: Visual Analog Scale; CI: confiance interval; T0: 1st postoperative day; T1: 7–15 day postoperative; T2: 3–6 month postoperative; T3: 12–month postoperative; T4: 18-month postoperative; T5: 24-month postoperative.

Assessment of the McGill scale^26^ results revealed that both the number of pain descriptors and the pain index were higher in the TG than in the FGG or the NFG only for the first postoperative period (T0).

This finding was not observed at other follow-up times (Tables 4 and 5).

**Table 4:** Group comparison of McGill pain descriptors^26^ per follow-up period.

McGill descriptor		Average	Median	Standard deviation	n	CI	p-value
T0	Non-fixation	2.33	2	1.20	21	0.51	0.002
	Glue	3.43	3	0.87	21	0.37	
	Tackers	4.52	5	0.93	21	0.40	
T1	Non-fixation	1.24	1	0.44	21	0.19	0.500
	Glue	1.33	1	0.48	21	0.21	
	Tackers	1.76	2	0.70	21	0.30	
T2	Non-fixation	0,05	0	0,22	21	0,09	0.317
	Glue	0.00	0	0.00	21	–	
	Tackers	0.00	0	0.00	21	–	
T3	Non-fixation	0.00	0	0.00	21	–	1,000
	Glue	0.00	0	0.00	21	–	
	Tackers	0.00	0	0.00	21	–	
T4	Non-fixation	0.00	0	0.00	21	–	1,000
	Glue	0.00	0	0.00	21	–	
	Tackers	0.00	0	0.00	21	–	
T5	Non-fixation	0.00	0	0.00	21	–	1,000
	Glue	0.00	0	0.00	21	–	
	Tackers	0.00	0	0.00	21	–	

CI: confiance interval; T0: 1st postoperative day; T1: 7–15 day postoperative; T2: 3–6 month postoperative; T3: 12–month postoperative; T4: 18-month postoperative; T5: 24-month postoperative.

**Table 5:** Group comparison of the McGill pain index^26^ per follow-up period.

McGill index		Average	Median	Standard deviation	n	CI	p-value
T0	Non-fixation	3.48	2	3.57	21	1.53	0.021
	Glue	4.29	4	2.24	21	0.96	
	Tackers	11.76	12	5.40	21	2.31	
T1	Non-fixation	1.24	1	0.44	21	0.19	1,000
	Glue	1.24	1	0.44	21	019	
	Tackers	1.76	2	0.70	21	0.30	
T2	Non-fixation	0.05	0	0.22	21	0.09	0.317
	Glue	0.00	0	0.00	21	–	
	Tackers	0.00	0	0.00	21	–	
T3	Non-fixation	0.00	0	0.00	21	–	1,000
	Glue	0.00	0	0.00	21	–	
	Tackers	0.00	0	0.00	21	–	
T4	Non-fixation	0.00	0	0.00	21	–	1,000
	Glue	0.00	0	0.00	21	–	
	Tackers	0.00	0	0.00	21	–	
T5	Non-fixation	0.00	0	0.00	21	–	1,000
	Glue	0.00	0	0.00	21	–	
	Tackers	0.00	0	0.00	21	–	

CI: confiance interval; T0: 1st postoperative day; T1: 7–15 day postoperative; T2: 3–6 month postoperative; T3: 12–month postoperative; T4: 18-month postoperative; T5: 24-month postoperative.

The descriptor most described was in 33% pain like sharp followed by pain like tenderness in 15.6%. There was no affective descriptor of pain in McGill scale in any period.

When the groups were compared in pairs at T0 (using Mann-Whitney U test), differences were found because pain descriptors were significantly higher in the TG than in the others. Pain descriptors were also significantly higher in the FGG than the NFG only in T0 (Tables 6 and 7).

**Table 6:** Between-group pairwise comparison of the McGill pain index^26^.

	Non-fixation/fibrin glue	Non-fixation/tackers	Glue/tackers
T0	0.021	<0.001	<0.001
T1	NS	NS	NS
T2	NS	NS	NS
T3	NS	NS	NS
T4	NS	NS	NS
T5	NS	NS	NS

NS: not significant; T0: 1st postoperative day; T1: 7–15 day postoperative; T2: 3–6 month postoperative; T3: 12–month postoperative; T4: 18-month postoperative; T5: 24-month postoperative.

**Table 7:** Between-group pairwise comparison of the McGill pain descriptors^26^.

	Non-fixation/fibrin glue	Non-fixation/tackers	Fibrin glue/tackers
T0	0.002	<0.001	<0.001
T1	NS	NS	NS
T2	NS	NS	NS
T3	NS	NS	NS
T4	NS	NS	NS
T5	NS	NS	NS

NS: not significant; T0: 1st postoperative day; T1: 7–15 day postoperative; T2: 3–6 month postoperative; T3: 12–month postoperative; T4: 18-month postoperative; T5: 24-month postoperative.

During the 2-year follow-up, no hernia recurrences were observed in any of the three groups.

## DISCUSSION

Inguinal hernia has a high prevalence in the population, affecting approximately 27% of men and 6% of women, and inguinal hernioplasty is one of the world’s most widely performed surgeries^8,14,18,19,28^. Bullen et al.^9^ conducted a meta-analysis and systematic review on this topic and reported that patients undergoing laparoscopic repair have a lower rate of both acute and chronic pain. In a publication by the Herniasurge Group^7,14,17^, it was shown that videolaparoscopic inguinal hernioplasty is less likely to produce chronic pain and, in addition, leads to faster return to usual activities^1,7,15,24,34^. Andresen et al.^1^, who aimed at comparing tackers and fibrin glue as mesh-fixation methods, suggested that the mesh fixation did not correlate with the onset of chronic pain. However, patients in the FGG experienced a lower rate of pain in the first postoperative days. In order to avoid bias, the design of this study established that the surgeon chosen for postoperative patient follow-up must not know the mesh-fixation technique, or whether the mesh had been fixated. During follow-up, no statistical difference was observed regarding the onset of chronic pain on VAS for a period of 24 months, regardless of the method used for mesh fixation, as shown in the systematic review conducted by Lederhuber et al.^24^. In an attempt to dissociate the direct relationship between tissue injury and pain and that in any painful experience, sensitive, emotional, and cognitive aspects can be a bias, the McGill scale was applied, which is one of the best instruments and the most used to characterize and discern the components affective, sensitive, and evaluative of pain. So, the patients were asked to point out, based on a list of pain descriptors, the type and the pain quality experienced. It was found that the ones in the TG had chosen more descriptors and a higher pain index, calculated for a period up to 15 days postoperatively (T0 and T1). Based on our results, we concluded that mesh fixation with a penetrating method (tackers) may cause a greater sensation of discomfort than other atraumatic methods such as glue fixation or mesh placement without fixation. Our results also revealed that patients in the FGG chose more pain descriptors and a higher pain index on the McGill scale than those in the NFG.

These results are in line with studies conducted by Reinpold et al.^31^ and reiterate that as far as pain is concerned, whether acute or chronic, the factors leading to increased patient sensitivity and perception of pain include not only direct nerve injury but also inflammatory reaction with acute healing around the tacker or around the point where glue was applied.

To provide an additional perspective on the onset of chronic postoperative pain, a group of patients was included in this study, to whom the mesh was not fixated but rather only placed after the anatomical repairs on the myopectineal orifice of Fruchaud were identified^12,13,15^. In this group of patients, both the number of pain descriptors and the McGill pain index^26^ were found to be lower than those observed in the other two groups (TG and FGG). Based on our results, we suggest that careful and accurate dissection, especially close to nerve trajectories, and observation of all the technical and anatomical aspects of the myopectineal orifice^12,13,15,32^ are essential steps to avoid the onset of chronic pain, with or without mesh fixation.

During the 24-month follow-up with no patients lost, no hernia recurrence was identified in any of the three groups, including the NFG, although our study has a limited casuistic the results were similar to those presented in studies published recently, and in line with the current trend toward no mesh fixation in TAPP repair of both indirect primary hernias (regardless of size) and direct hernias measuring up to 3 cm. And it is worth underlining that the recurrence in non-fixation TAPP may be due to clamshelling and dislocation of the mesh, while in TAPP with fixation (glue or tackers) prevent mesh folding. It may be a crucial factor preventing recurrences; thus, sufficient dissection of preperitoneal space is of utmost attention.

During this follow-up, 7.94% (n=5) developed seroma postoperatively and were all treated conservatively, and their symptoms disappeared in up to 6 months (T2) after surgery. This result is consistent with the literature worldwide^22,29^ and did not affect the onset of any pain symptoms.

## CONCLUSION

In our study, the method chosen for mesh fixation in TAPP inguinal hernia repair, whether tacker or glue fixation, did not influence the onset of chronic pain. However, it was noted that tackers may increase a patient’s sensitivity to pain in the inguinal region. Mesh placement without fixation led to the same pain and recurrence outcomes as those found in mesh-fixation techniques. Consequently, non-fixation has become an alternative therapy worth considering. In this study, no cases of hernia recurrence were found during follow-up.
